# Supplementary material for: Diaphorin, a Polyketide Produced by a Bacterial Symbiont of the Asian Citrus Psyllid, Inhibits the Growth and Cell Division of Bacillus subtilis but Promotes the Growth and Metabolic Activity of Escherichia coli
Source: Microbiol Spectr. 2022 Jul 27;10(4):e01757-22. doi: 10.1128/spectrum.01757-22 (PMC9430481; doi:10.1128/spectrum.01757-22)
Supplement: Supplemental file 1 — Supplemental material. Download spectrum.01757-22-s0001.pdf, PDF file, 2.9 MB [file spectrum.01757-22-s0001.pdf]

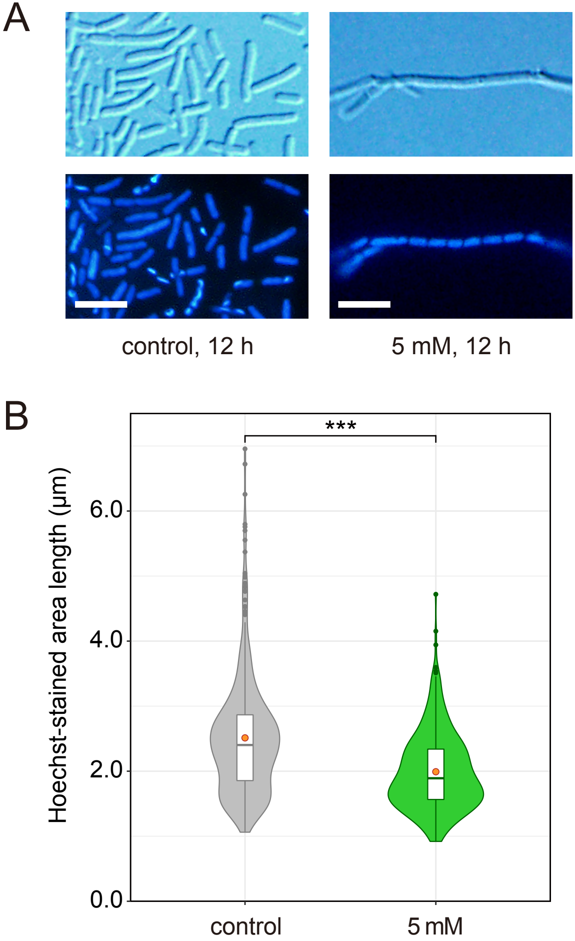

Figure S1. Chained *B. subtilis* cells caused by diaphorin treatment. (A) DIC and fluorescence images of Hoechst-stained *B. subtilis* cells after cultivation for 12 h in a medium containing 0 or 5 mM of diaphorin. Bars, 5 µm. (B) Lengths of Hoechst-stained nucleoid areas in *B. subtilis* cells cultured in a medium containing 0 (gray; n = 400) or 5 mM diaphorin (green; n = 400) for 12 h. Violin plots showing kernel density estimation are overlaid with box plots showing median, quartiles, minimum, maximum, and outliers. Orange dots represent the means.
